# Comparative Effects of High-Intensity Interval Training and Low-Intensity Steady-State Exercise on Anthropometric Outcomes and Psychophysical Well-Being: A Pilot Study

**DOI:** 10.3390/jfmk11010088

**Published:** 2026-02-20

**Authors:** Felice Di Domenico, Giovanni Esposito, Sara Aliberti, Rosario Ceruso, Gaetano Raiola

**Affiliations:** 1 Research Center of Physical Education and Exercise, Pegaso University, 80143 Naples, Italy; flcdidomenico@gmail.com (F.D.D.); giovanniesposito410@gmail.com (G.E.); saaliberti@unisa.it (S.A.); gaetano.raiola@unipegaso.it (G.R.); 2 Department of Neuroscience, Biomedicine and Movement, University of Verona, 37134 Verona, Italy

**Keywords:** interval training, continuous aerobic exercise, body composition, somatotype, ISAK anthropometry, skinfold assessment, training adaptations

## Abstract

**Background:** Comparative evidence for the effectiveness of High-Intensity Interval Training (HIIT) and Low-Intensity Steady-State (LISS) exercise derived from real-world settings using standardized anthropometric assessments and subjective perception measures remains limited. This pilot study aimed to compare the effects of 12 weeks of HIIT and LISS on anthropometric outcomes assessed through ISAK measurements and on post-intervention subjective perceptions. **Methods**: Twenty-four physically active adults (*n* = 12 HIIT; *n* = 12 LISS) completed a 12-week supervised training program with three sessions per week. Exercise intensity was monitored by heart rate (HIIT: 80–95% HRmax; LISS: 60–70% HRmax). Anthropometric measurements were performed according to ISAK guidelines at baseline and after 4, 8, and 12 weeks. Subjective perceptions were assessed post-intervention using a structured questionnaire. Data were analyzed using parametric or non-parametric tests, as appropriate. **Results**: Both HIIT and LISS showed significant reductions over time in body mass, BMI, fat mass, and waist and hip circumferences (*p* < 0.001), with no between-group differences for the primary endpoint. Endomorphy decreased and ectomorphy increased significantly in both groups. A significant group × time interaction was observed for muscle mass (*p* < 0.001), favoring preservation or slight increases in HIIT. Post-intervention, psychological well-being was higher in LISS (*p* = 0.002), whereas perceived physical performance improvements were greater in HIIT (*p* = 0.002–<0.001). **Conclusions**: In a real-world personal training context, HIIT and LISS produced comparable improvements in body composition while eliciting different perceptual responses, supporting individualized exercise prescription.

## 1. Introduction

Regular physical activity is one of the main determinants of psychophysical well-being, with well-documented effects on mental health, emotional regulation, and quality of life [1]. In recent years, increasing attention has been devoted to brief and frequent forms of cardiovascular exercise, such as structured walking sessions, moderate circuits, or short bouts of high-intensity activity, which appear effective in eliciting perceived benefits even in non-athletic populations [2,3].

Cardiorespiratory training represents one of the most effective and widely adopted strategies to improve body composition, aerobic capacity, and overall health in adults [1,4]. However, different exercise and movement experiences may induce distinct adaptations in terms of effectiveness, sustainability, and perceptual responses, as subjective psychophysical outcomes are influenced by individual characteristics and the interaction between motor performance and self-perceived well-being [3,5]. Among the most extensively studied modalities, High-Intensity Interval Training (HIIT) and Low-Intensity Steady-State (LISS) exercise represent two contrasting approaches that have received growing scientific attention.

HIIT, characterized by short bouts of high-intensity exercise alternated with recovery periods, has been associated with significant improvements in aerobic capacity and body composition, despite reduced time commitment compared with traditional continuous training [6]. Several studies have shown that HIIT can elicit substantial metabolic and neuromuscular adaptations, leading to improvements in body composition and physical performance across different populations, including physically active adults and athletes involved in intermittent sports [7,8,9]. A recent umbrella review further suggests that, although both modalities are effective, interval training may confer a small advantage in fat mass reduction compared with continuous exercise, despite considerable methodological heterogeneity across studies [10].

From a physiological perspective, the potential benefits of HIIT are attributed to enhanced stimulation of signaling pathways involved in mitochondrial biogenesis and to increased post-exercise oxygen consumption (EPOC), which contributes to higher overall energy expenditure [3,11]. In addition, studies conducted in overweight adults have shown that structured HIIT protocols may improve metabolic biomarkers, mitochondrial enzyme activity, and insulin sensitivity even over relatively short intervention periods [12].

Conversely, LISS consists of continuous exercise performed at low-to-moderate intensity for prolonged durations. Although often considered less effective than HIIT for fat loss, LISS is nonetheless associated with meaningful improvements in aerobic capacity and lipid metabolism efficiency [13]. Moreover, due to its lower intensity, LISS is frequently linked to greater adherence, long-term sustainability, and lower perceived exertion—factors that are particularly relevant in non-competitive fitness contexts [14]. Longitudinal studies in adult and older populations have also reported significant positive effects on perceived well-being even in the absence of high-intensity stimuli [15].

Despite evidence supporting both methods, important gaps remain in the literature, including substantial heterogeneity in training protocols, limited control of internal training load, and scarce integration of standardized anthropometric assessments—such as those proposed by the International Society for the Advancement of Kinanthropometry (ISAK)—with subjective well-being evaluations. Furthermore, many studies rely on non-uniform methodologies (e.g., bioelectrical impedance, BMI, or non-standardized circumferences), limiting comparability across investigations.

Psychological responses to exercise also remain relatively underexplored, despite growing evidence that both HIIT and LISS may positively influence stress, anxiety, and mood, with individual variability related to intensity, duration, self-efficacy, and personal preferences [16]. Previous research highlights the importance of assessing not only physiological outcomes but also subjective perceptions, which represent key predictors of adherence, motivation, and psychological response to physical activity [17,18]. In this perspective, movement influences psychophysical processes through perceptual, emotional, and cognitive mechanisms, underscoring the need for integrated evaluation of physical and psychological dimensions in exercise and sport contexts [19]. Subjective perceptions of well-being and physical activity have been shown to represent reliable and functional indicators in applied, educational, and training-oriented contexts, as they reflect the integration of physical exertion, psychophysical learning processes, and individual experience within structured exercise programs [20].

In light of these considerations, there is a need for methodologically rigorous and replicable investigations examining the comparative effects of HIIT and LISS on anthropometric variables and subjective perceptions of well-being within real-world applied contexts, such as supervised personal training. Accordingly, the present study was designed as a pilot investigation aimed at assessing the feasibility of the experimental protocol, exploring participant response patterns, and generating preliminary data to inform future controlled trials.

The primary objective of this pilot study was to compare the effects of 12 weeks of HIIT and LISS on changes in fat mass estimated through ISAK skinfold measurements. Secondary objectives were to: (a) describe changes in somatotype, BMI, and body circumferences; (b) compare post-intervention perceptions of psychophysical well-being and perceived performance improvements; and (c) explore potential relationships between anthropometric changes and subjective perceptions. Given the pilot nature and applied context of the study, between-group comparisons were interpreted in an exploratory framework. Consistent with these aims, a quasi-experimental parallel-group design was adopted within a supervised personal training setting, with non-randomized allocation reflecting real operational conditions. Anthropometric assessments were conducted at multiple time points to describe temporal trends in body composition variables, while subjective perceptions were collected post-intervention for descriptive and exploratory purposes.

## 2. Materials and Methods

### 2.1. Study Design

This study was conducted as a pilot quasi-experimental parallel-group study aimed at exploring the effects of two different cardiovascular training protocols (High-Intensity Interval Training, HIIT, and Low-Intensity Steady-State exercise, LISS) in a supervised personal training setting. The intervention lasted 12 weeks and included repeated assessments of anthropometric variables throughout the study period (T0, T1, T2, and T3). Participant allocation to the two training protocols was non-randomized and determined by organizational constraints and by the type of training program already undertaken, in order to reflect real-world operational conditions. Specifically, allocation followed the pre-existing training pathway at the facility: participants already enrolled in HIIT-like sessions were assigned to the HIIT group, whereas those enrolled in continuous aerobic sessions were assigned to the LISS group. Given the pilot nature of the study, the results were interpreted using a descriptive and exploratory approach.

### 2.2. Participants

Adult men and women participating in supervised, non-competitive personal training programs at a fitness center were recruited for the study. All participants had been engaged in supervised training for at least 12 months prior to enrolment, typically consisting of combined resistance and aerobic exercise sessions. Inclusion criteria were: Age between 20 and 35 years; regular participation in supervised training programs for at least 12 months; and absence of medical contraindications to moderate- or high-intensity exercise. Exclusion criteria included acute musculoskeletal injuries within the previous three months, diagnosed cardiovascular or metabolic conditions contraindicating moderate-to-vigorous exercise, and any condition limiting full participation in the training protocols. All participants were informed about the study aims and procedures and provided written informed consent prior to participation.

### 2.3. Ethics Approval and Informed Consent

The study was conducted in accordance with the Declaration of Helsinki. The protocol was approved within the research project entitled “Psychophysical perception and body awareness in school and sports contexts through observational and non-invasive tools” by the Ethics Committee of Pegaso University (Prot./E 004726, dated 15/07/2025). All participants were fully informed about the aims, procedures, and potential risks of the study and provided written informed consent prior to participation. Participant confidentiality and data protection were ensured throughout the study.

### 2.4. Training Protocols

The intervention lasted 12 weeks and consisted of three training sessions per week, each lasting approximately 50–60 min. All sessions were supervised by qualified exercise professionals, who ensured correct exercise execution and continuously monitored training load and protocol-specific intensity. The HIIT and LISS protocols were not matched for total energy expenditure but were designed to reflect training modalities commonly used in real-world personal training practice, in line with an applied and pragmatic comparison.

### 2.5. Intensity Monitoring

Exercise intensity was monitored during all training sessions using a Polar H9^®^ chest-strap heart rate monitor (Polar Electro Oy, Kempele, Finland), allowing continuous recording of heart rate and verification of adherence to the prescribed internal load ranges. Target intensity zones were defined as follows:

HIIT: 80–95% of maximum heart rate (HRmax);

LISS: 60–70% of HRmax.

HRmax was estimated using the age-predicted equation (HRmax = 220 − age), and values were entered into the Polar Flow system to define individual training zones.

Heart rate data (Table 1), including beats per minute (bpm), percentage of HRmax, and time spent in predefined intensity zones, were synchronized via the Polar Flow application at the end of each training session.

### 2.6. HIIT Protocol

The HIIT protocol consisted of training sessions lasting approximately 50 min, including warm-up and cool-down phases. The main phase involved the execution of high-intensity circuits composed of six multi-joint exercises (e.g., squats, lunges, mountain climbers, jumping jacks, push-ups, and burpees), selected to engage large muscle groups.

Each exercise was performed for 30 s at high intensity, followed by 30 s of active recovery. The circuit was repeated for four rounds, with 2 min of passive recovery between rounds. Target exercise intensity during the work intervals ranged between 85% and 95% of estimated maximum heart rate and was monitored in real time using a heart rate monitor.

Training load progression was implemented gradually over the 12-week intervention, primarily through increased execution intensity and sustained maintenance of heart rate within the prescribed target zones. In addition, exercise complexity was progressively adjusted while keeping the temporal structure of the protocol unchanged. A representative HIIT session is presented in Table 2.

### 2.7. LISS Protocol

The LISS protocol consisted of continuous aerobic training sessions lasting approximately 50 min. Sessions were performed using a treadmill, elliptical trainer, stationary bicycle, or stair climber, depending on equipment availability and participant preference. However, session duration and target intensity were standardized across participants to ensure comparable internal load. Exercise intensity was maintained within 60–70% of estimated maximum heart rate (HRmax) and continuously monitored using a heart rate monitor.

Each session included:

- 5 min of warm-up (50–60% HRmax);
- 40 min of continuous exercise;
- 5 min of cool-down.

Progression of the LISS protocol was achieved through slight increases in treadmill speed or incline in order to maintain heart rate within the prescribed target range throughout the intervention period. A representative LISS session is reported in Table 3.

Training progression across the 12-week intervention is summarized in Table 4; progression was primarily achieved by maintaining heart rate within the prescribed target zones and, when necessary, by slight adjustments in exercise execution intensity or workload.

### 2.8. Equipment

The following equipment was used during the training sessions: treadmill, stationary bicycle, elliptical trainer, and stair climber; jump rope; kettlebells; battle ropes; TRX suspension system; barbells, dumbbells, and adjustable benches; medicine balls, Swiss balls, and ab rollers; parallel bars and pull-up bars; elastic resistance bands of varying intensities; and cable machines. This equipment was primarily used in the HIIT group, while the LISS group mainly utilized the treadmill, stationary bicycle, and elliptical trainer.

### 2.9. Nutritional Control

To minimize potential confounding effects related to dietary changes, participants were instructed to maintain their habitual dietary patterns throughout the intervention and to avoid initiating any nutritional programs aimed at modifying body weight or body composition. Dietary intake was monitored using a 3-day food diary (two weekdays and one weekend day), collected at baseline (T0) and at the end of the intervention (T3). Food diaries were used to estimate daily energy intake and macronutrient distribution.

### 2.10. Anthropometric Measurements

Anthropometric assessments were conducted in accordance with the guidelines of the International Society for the Advancement of Kinanthropometry (ISAK) to ensure measurement standardization and replicability. All measurements were performed by a single ISAK-certified assessor using calibrated anthropometric instruments. Assessments were carried out at four time points: baseline (T0), after 4 weeks (T1), after 8 weeks (T2), and at the end of the 12-week intervention (T3). Measurements were performed under the same operational conditions and following standardized procedures at each time point.

The following variables were assessed: stature, body mass, body circumferences (including waist and hip), and skinfold thicknesses (triceps, subscapular, biceps, iliac crest, supraspinale, abdominal, thigh, and calf). Each measurement was taken twice, and the mean value was used for statistical analysis. Percentage body fat was estimated from skinfold measurements using standardized equations.

Based on the anthropometric data, Body Mass Index (BMI), waist-to-hip ratio (WHR), and somatotype according to the Heath–Carter method were calculated. These indices were used for descriptive and comparative purposes to characterize morphological changes over time and potential differences between the two training protocols.

### 2.11. Subjective Well-Being Questionnaire

Subjective perceptions related to psychophysical well-being, perceived performance improvement, and body composition were assessed using an ad hoc structured questionnaire administered exclusively at the end of the intervention (T3). The relevant subsection has been revised to explicitly state that the instrument was not previously validated and that the findings should therefore be interpreted as exploratory trends; this limitation has also been acknowledged in the Discussion. Ad hoc instruments are frequently used in pilot and exploratory investigations, provided that findings are interpreted descriptively and with appropriate caution, particularly in applied real-world contexts. The questionnaire comprised three main domains, each including two items rated on a 5-point Likert scale.

Psychological well-being:

1. I feel psychologically better compared with before the intervention.
2. I perceived an increase in my motivation during the training program.

Physical/functional well-being:

1. I perceive an improvement in my overall physical well-being.
2. I feel more capable and functional in performing daily activities.

Perceived performance improvement:

1. I feel that my overall physical performance has improved.
2. I perceive myself as stronger, faster, or more resistant compared with before the intervention.

Given the post-intervention-only administration and the exploratory nature of the instrument, subjective perception data were analyzed for descriptive and exploratory purposes and interpreted with caution. Accordingly, the resulting findings should be considered preliminary and descriptive indications rather than robust or clinically validated psychological outcomes.

### 2.12. Statistical Analysis

Statistical analyses were performed using JASP (version 0.95.4). The primary endpoint of the study was defined as the change in percentage body fat estimated via skinfold measurements. Secondary endpoints included changes in key anthropometric variables (body mass, circumferences, BMI, and somatotype) and subjective perceptions of well-being and perceived improvement assessed at the end of the intervention.

The distribution of continuous variables was assessed using the Shapiro–Wilk test. Anthropometric data were analyzed across three levels of comparison: (i) within-group pre–post changes, (ii) between-group comparisons, and (iii) temporal trends across the intervention period.

For longitudinal analysis of anthropometric measures collected at four time points (T0, T1, T2, and T3), a repeated-measures analysis of variance (ANOVA) was performed with group (HIIT vs. LISS) as the between-subject factor and time as the within-subject factor. When the assumption of sphericity was violated, the Greenhouse–Geisser correction was applied. Where appropriate, post hoc comparisons were conducted to explore intra-group and inter-group differences, with adjustment for multiple comparisons.

Pre–post changes within each group were further described by calculating absolute and percentage differences between T0 and T3. Intervention effects were quantified using effect size estimates and corresponding 95% confidence intervals, where applicable. Effect sizes for between-group comparisons were calculated using Cohen’s d (mean change difference divided by the pooled standard deviation of change scores). Values were interpreted as small, moderate, and large according to conventional thresholds. Cohen’s d values were interpreted as small (0.2), medium (0.5), and large (0.8) according to Cohen [21].

Subjective perception data, collected exclusively at T3, were analyzed for descriptive and exploratory purposes. Between-group comparisons for perceptual variables were performed using appropriate tests for ordinal or categorical data, depending on response distribution. Given the pilot nature of the study, analyses of secondary endpoints and perceptual data were interpreted within an exploratory framework. Statistical significance was set at *p* < 0.05.

Given the non-randomized allocation and pilot nature of the study, inferential statistics were used in an exploratory manner, and results should be interpreted with caution.

## 3. Results

### 3.1. Baseline Characteristics of the Participants

Baseline anthropometric characteristics of the participants (T0) are reported in Table 5. No statistically significant differences were observed between the HIIT and LISS groups for any of the baseline variables (all *p* > 0.05), indicating comparable initial characteristics.

### 3.2. Within-Group Pre–Post Changes

Descriptive trends in the main anthropometric variables across the intervention (T0–T3) are reported in Table 6. Mean values for body mass, BMI, and circumferences are presented for both groups at each time point. Inferential within-group and between-group analyses are reported in Section 3.3, Section 3.4, Section 3.5 and Section 3.6.

### 3.3. HIIT: T0–T3 Comparison

Within-group T0–T3 comparisons for the HIIT group are reported in Table 7. Significant reductions were observed in body mass, several skinfold thicknesses, and waist and hip circumferences. Estimated muscle mass did not show a significant decrease over the intervention period.

Overall, the HIIT protocol induced significant improvements in most adiposity-related variables, while muscle mass indices remained stable, indicating a favorable body composition response over the intervention period.

### 3.4. LISS: T0–T3 Comparison

Within-group T0–T3 comparisons for the LISS group are reported in Table 8. Significant reductions were observed in body mass, several skinfold thicknesses, and waist and hip circumferences. Estimated muscle mass remained overall stable throughout the intervention period.

Overall, the LISS protocol resulted in significant improvements in adiposity-related variables, while muscle mass indices were largely preserved over the intervention period.

### 3.5. Between-Group Differences

Between-group comparisons of Δ(T3–T0) are reported in Table 9. No statistically significant differences were detected between HIIT and LISS for any variable (all *p* > 0.05). Effect sizes (Cohen’s d) are provided to support interpretation of the magnitude of group differences.

### 3.6. Time Effects

Mixed-design ANOVA (Table 10) revealed significant decreases in body mass, BMI, and fat mass over the 12-week intervention period (*p* < 0.001), with no significant differences between the HIIT and LISS groups and no significant group × time interactions.

Regarding muscle mass, the main effect of time was not significant (*p* = 0.840); however, a significant group × time interaction emerged (*p* < 0.001). This result suggests that muscle mass trajectories differed between groups, with a favorable trend in the HIIT group, which showed a slight increase, compared with the LISS group, which exhibited a slight decrease.

Somatotype components showed distinct patterns over time. Endomorphy decreased significantly across the intervention period (*p* < 0.001), indicating a reduction in the adiposity component, with no differences between groups. Ectomorphy also increased significantly over time (*p* < 0.001), reflecting a relative increase in linearity, again without between-group differences or significant interactions. Mesomorphy did not show significant changes over time (*p* = 0.828) nor differences between groups.

Overall, the ANOVA findings indicate significant time-related improvements in adiposity-related variables in both training modalities, with largely similar responses between groups, except for the differential muscle mass trajectory.

### 3.7. Analysis of Psychophysical Well-Being Perception

Analysis of the post-intervention questionnaire (Table 11) using chi-square tests revealed significant between-group differences for three of the six evaluated items. Within the psychological domain, the item “feeling psychologically better” showed a significantly different distribution between groups, χ^2^(2) = 12.90, *p* = 0.002, with the LISS group reporting higher perceived psychological well-being compared with the HIIT group.

Within the performance domain, the HIIT group reported significantly greater improvements both in perceived overall physical performance, χ^2^(3) = 15.11, *p* = 0.002, and in perceived strength, speed, or endurance, χ^2^(3) = 20.67, *p* < 0.001. In both cases, effect sizes were medium-to-large (Cramér’s V = 0.46) and large (Cramér’s V = 0.54), respectively.

The remaining items related to motivation, general physical well-being, and daily functional capacity did not show significant differences between the HIIT and LISS groups (all *p* > 0.11), indicating comparable perceived improvements in these domains across both training protocols. Given the exploratory nature of the questionnaire and its post-intervention administration, these findings should be interpreted as preliminary.

## 4. Discussions

The present pilot study compared the effects of 12 weeks of High-Intensity Interval Training (HIIT) and Low-Intensity Steady-State (LISS) exercise, conducted in a supervised personal training setting, on body composition and subjective perceptions of well-being and improvement. The main findings indicate that both training protocols were associated with reductions in body fat percentage over time, with no statistically significant between-group differences for the primary endpoint, while differences emerged in subjective perceptions assessed at the end of the intervention. Therefore, these findings should be considered preliminary and hypothesis-generating rather than confirmatory.

### 4.1. Summary of Main Findings

#### 4.1.1. Reductions in Body Mass and Body Fat

Mixed-design ANOVA revealed a significant main effect of time for body mass, BMI, and fat mass, indicating a general improvement independent of training modality. Paired analyses confirmed significant reductions in both groups across the main adiposity-related variables.

Pre–post comparisons further indicated that HIIT was associated with a greater reduction in fat mass (−2.69 kg) compared with LISS (−1.69 kg), with a medium-to-large effect size (d = −0.58), although the between-group comparison did not reach statistical significance (*p* = 0.13). In addition, triceps, iliac crest, and abdominal skinfolds consistently showed more pronounced reductions in the HIIT group.

These findings are consistent with the existing literature indicating that HIIT promotes body fat reduction through a range of physiological adaptations that should be considered when designing physical activity and exercise training programs. The high intensities typical of HIIT induce a greater homeostatic perturbation than moderate-intensity continuous exercise, resulting in a prolonged elevation of post-exercise metabolism [6,21]. From an applied perspective, excess post-exercise oxygen consumption (EPOC) represents a key mechanism for optimizing fat loss in time-efficient physical activity programs, a frequent constraint in fitness and wellness contexts, as it contributes to increased total energy expenditure in the hours following exercise. Evidence shows that the magnitude of EPOC is strongly influenced by exercise intensity and is greater following high-intensity protocols compared with moderate-intensity continuous exercise [11,24,25].

In particular, High-Intensity Interval Training has been associated with a prolonged elevation in post-exercise metabolism, which may enhance lipid oxidation and contribute to fat mass reduction despite a lower total training volume [6,26]. Moreover, HIIT appears to promote increased mobilization of fatty acids from adipose tissue, particularly in the abdominal region. This mechanism may help explain the more pronounced reductions observed in abdominal skinfolds and waist circumference following high-intensity exercise [27,28].

It should also be noted that, following high-intensity exercise, the body tends to preferentially rely on fat oxidation during the recovery phase. Recent studies have shown that HIIT induces significant improvements in skeletal muscle oxidative capacity through increased activity of oxidative enzymes [29,30]. This suggests that HIIT not only reduces fat mass but also contributes to enhanced overall metabolic efficiency.

Functional HIIT protocols incorporating multi-joint movements, plyometric exercises, and accelerations elicit greater recruitment of type II muscle fibers, higher mechanical tension, and increased neuromuscular demand compared with LISS. These stimuli support the maintenance or even slight increases in lean mass, even within predominantly cardiovascular training programs [31,32]. This aspect is particularly relevant given that the goal of exercise training extends beyond weight loss to include the preservation of physical function and performance in daily and sport-related movements.

Numerous studies have reported that HIIT can induce adaptations comparable or superior to those observed with LISS, despite lower overall training volumes [3,7]. This represents an important advantage for physical activity programs targeting populations with limited time availability, a central issue in the promotion of physical activity within the general population.

Importantly, the primary endpoint of the study—change in fat mass estimated from skinfold measurements—did not significantly differ between HIIT and LISS, indicating that both training modalities were similarly effective in reducing adiposity within the limits of this pilot investigation.

#### 4.1.2. Muscle Mass

A significant group × time interaction was observed for muscle mass, indicating different adaptation patterns between the two training protocols. Specifically, the HIIT group showed a slight increase in muscle mass (+0.47 kg), whereas the LISS group exhibited a small reduction (−0.16 kg). Although the between-group comparison did not reach conventional statistical significance (*p* = 0.06), the associated effect size was large (Cohen’s d = 0.81), suggesting a meaningful difference from an applied perspective.

These findings indicate that HIIT may be more effective in preserving muscle mass during a fat-loss-oriented training intervention. A plausible explanation is that HIIT protocols incorporating functional, multi-joint movements impose greater mechanical tension, higher neuromuscular demand, and increased recruitment of fast-twitch muscle fibers compared with continuous low-intensity exercise. Such stimuli are known to elicit a stronger acute anabolic response and to support the maintenance of lean mass even within predominantly cardiovascular training programs [31,32].

#### 4.1.3. Circumferences and Anthropometric Indices

Both training protocols produced significant improvements in several anthropometric indicators, particularly waist circumference (HIIT: −6.89 cm; LISS: −5.72 cm) and the conicity index, with a more favorable trend observed in the HIIT group (*p* = 0.06, d = −0.47). These findings are highly relevant from a physiological, preventive, and applied perspective. Waist circumference is recognized as one of the most sensitive markers of cardiometabolic risk [33]. Reductions greater than 5 cm, as observed in both groups, are considered clinically meaningful and are associated with improvements in insulin sensitivity, blood pressure, and lipid profile [34,35].

The literature indicates that reductions in visceral adipose tissue (VAT) may occur even in the absence of marked changes in total body mass, making waist circumference a key indicator of the effectiveness of an exercise intervention. In parallel, the decrease in the conicity index, with a more pronounced trend in the HIIT group, suggests a reduction in central fat distribution and a lower accumulation of visceral abdominal adiposity. This index, originally proposed by Valdez [36], is strongly associated with cardiovascular risk parameters such as triglycerides, LDL cholesterol, and insulin resistance.

Although between-group differences did not reach statistical significance (p > 0.05), the presence of a moderate effect size suggests that HIIT may be more effective in promoting abdominal fat remodeling. Overall, these results indicate that both HIIT and LISS are valid strategies for cardiometabolic prevention interventions, while HIIT may represent a particularly efficient approach when the primary goal is the reduction of abdominal fat within shorter time frames or with lower overall training volume. These findings are consistent with recent meta-analyses reporting similar or greater reductions in visceral fat following HIIT compared with LISS protocols, despite a lower total exercise duration [7,26].

#### 4.1.4. Somatotype

The present findings confirm that the observed changes in somatotype were primarily driven by reductions in the adiposity component rather than by substantial modifications of muscular or skeletal structural components. With respect to endomorphy, mixed-design ANOVA revealed a significant main effect of time (*p* < 0.001), with no significant effects of group or group × time interaction. Both HIIT and LISS therefore induced significant reductions in the endomorphic component.

This pattern, further supported by paired *t*-test results showing significant within-group reductions and by the absence of between-group differences in independent comparisons, reflects an overall decrease in fat mass, consistent with the skinfold and fat mass data. The lack of significant differences between HIIT and LISS suggests that both protocols are effective in reducing adiposity, although HIIT showed a tendency toward a greater fat mass reduction (d = −0.58).

Mesomorphy did not exhibit significant main effects of time, group, or interaction in the mixed ANOVA. This finding can be interpreted in light of the muscle mass data collected over time: both groups largely maintained muscle mass (non-significant paired *t*-tests), and the between-group comparison showed a trend favoring HIIT (Δ +0.63 kg; *p* = 0.06; d = 0.81), without translating into significant changes in mesomorphy. This is consistent with the conceptual basis of the Heath–Carter somatotype, whereby mesomorphy reflects not only muscle mass but also skeletal robustness and proportionality, characteristics that typically require longer intervention periods or more specific strength-oriented stimuli to change meaningfully.

Ectomorphy showed a significant main effect of time in the mixed ANOVA (*p* < 0.001), with no significant group or interaction effects, indicating a similar increase in both HIIT and LISS. This increase can be interpreted as an enhancement of body linearity resulting from fat mass loss and reductions in both central and peripheral circumferences, particularly in the abdominal region.

These findings are consistent with previous studies on aerobic training, especially high-intensity modalities, which primarily induce adaptations related to fat mass reduction, whereas structural components generally require targeted resistance training and longer periodization to undergo substantial change [37,38]. The observed improvements in body composition are also in line with evidence from recent meta-analyses confirming that both HIIT and LISS can induce significant reductions in fat mass and BMI in healthy adults [7,39]. In particular, these reviews highlight that exercise intensity, rather than total training volume, often plays a key role in modulating fat loss, which may explain the greater tendency of HIIT to elicit faster and more pronounced changes.

The tendency of HIIT to preserve or even slightly increase lean mass is further supported by studies conducted in physically active populations, in which high-intensity protocols—especially those including multi-joint and functional exercises—induce greater neuromuscular stimulation compared with continuous aerobic exercise [32,40]. Similarly, the substantial reductions in abdominal circumferences observed in the present study are comparable to those reported in HIIT interventions involving individuals with overweight or central adiposity [26]. The observation of comparable effects in normal-weight or physically active individuals, as in the present study, suggests that HIIT may represent an effective strategy not only for weight management but also for optimizing metabolic health.

#### 4.1.5. Post-Intervention Perceptions of Psychophysical Well-Being and Performance

The analysis of participants’ subjective perceptions provided complementary information to the anthropometric outcomes, contributing to a more comprehensive understanding of the psychological, physical, and performance-related effects of the HIIT and LISS protocols. Chi-square analyses revealed significant differences in specific domains, suggesting that the two training programs may differentially influence subjective perceptions.

In particular, the psychological domain showed a significant between-group difference, with participants in the LISS group reporting higher levels of perceived psychological improvement. This finding is consistent with previous literature attributing continuous, moderate-intensity aerobic exercise to a greater capacity to induce stable states of relaxation, stress reduction, and improved affective responses, potentially due to lower sympathetic nervous system activation and higher perceived tolerability [41,42]. It is plausible that the continuous and less demanding nature of LISS fosters a stronger sense of control and subjective well-being compared with the intensive efforts required by HIIT protocols.

Conversely, domains related to perceived physical performance improvements showed significant differences favoring the HIIT group. Participants engaged in high-intensity training reported greater perceptions of strength, speed, endurance, and overall physical performance improvement. These results align with evidence indicating that HIIT, through its pronounced neuromuscular and metabolic stimuli, is particularly effective in enhancing anaerobic capacity, power output, and tolerance to high-intensity effort [43,44]. The higher perceived performance self-efficacy observed in the HIIT group may be explained by repeated exposure to highly challenging tasks, which facilitate mastery experiences—an established mechanism for strengthening perceived self-efficacy [45].

With regard to physical and functional aspects, such as general physical well-being and perceived daily functional capacity, no significant differences were observed between groups. This suggests that both protocols, despite differing in intensity, exert comparable effects on participants’ perceived physical condition. This convergence is consistent with studies showing that different exercise modalities, when practiced regularly, can produce similar benefits in perceived physical well-being and quality of life [46,47].

Overall, the findings highlight an interesting divergence between the two training modalities:

- LISS appears more effective in promoting subjective psychological well-being;
- HIIT appears more effective in enhancing perceived physical performance and capacity.

This distinction suggests that the selection of a training protocol should not be guided solely by anthropometric or physiological goals but also by the type of psychological adaptation desired. Differences in subjective perception indicate that training modality can shape the overall exercise experience and bodily awareness. In this regard, training approaches characterized by high neuromuscular activation have been associated with enhanced performance perception and motor awareness, even in the absence of marked physiological changes [48]. It should be noted that the way individuals interpret and manage the cognitive and emotional demands associated with physical exercise is inherently subjective. In sport and exercise contexts, psychological factors such as mental pressure, emotional regulation, and performance-related anxiety can significantly influence both objective performance outcomes and subjective experience. Specifically, the ability to regulate cognitive tension and maintain a focused, rational approach to motor execution has been associated with greater execution stability and improved psychophysical adaptation to effort. Previous research suggests that effective emotional and attentional control may facilitate more efficient motor responses and enhance perceived competence during physically demanding tasks [49].

In this perspective, the higher performance-related self-perceptions reported by the HIIT group may reflect not only physiological adaptations but also a greater exposure to cognitively and emotionally challenging tasks, which could foster improved self-regulation skills and heightened awareness of physical capabilities. Conversely, the more stable and predictable structure of LISS may promote psychological comfort and emotional balance, thereby enhancing perceived well-being. These findings highlight the importance of considering individual psychological responses when prescribing exercise modalities and reinforce the value of integrating psychophysical dimensions into training program design. For exercise science professionals, these findings support the importance of incorporating motivational factors and perceptual responses into the design of individualized training programs, thereby optimizing both effectiveness and long-term adherence.

### 4.2. Limitations and Practical Implications

The present study has several limitations typical of pilot investigations, whose primary aim is to assess feasibility and generate preliminary data for larger-scale research. First, the small sample size (*n* = 24) limits statistical power and may have prevented the detection of between-group differences despite the presence of practically meaningful trends. Second, the non-randomized group allocation may have introduced selection bias related to baseline participant characteristics, although this approach reflects the real-world conditions of applied personal training settings. Additionally, the lack of energy expenditure matching between protocols, while representative of real-world practice, limits the causal interpretation of the observed differences. Variations in total training volume and caloric expenditure between HIIT and LISS sessions may have influenced the magnitude of fat mass reduction in each group. Although HIIT is characterized by higher intensity and potential post-exercise metabolic effects, differences in total work performed could partially explain the adiposity-related outcomes. Therefore, it cannot be excluded that some of the observed trends in fat mass reduction reflect disparities in overall energy expenditure rather than the training modality per se.

Additionally, the use of an ad hoc, non-validated questionnaire administered exclusively at post-intervention represents a further limitation. Although the findings were interpreted cautiously as exploratory trends, the absence of prior validation limits the strength and generalizability of the psychophysical perception outcomes.

Despite these limitations, the findings provide useful insights for applied contexts. A combined and individualized programming approach integrating both HIIT and LISS may allow practitioners to exploit the complementary benefits of the two modalities, thereby optimizing both physiological and psychological adaptations. The significant reductions in waist circumference observed in both groups further emphasize the role of structured exercise in reducing cardiometabolic risk, reinforcing physical activity as a key preventive intervention.

The differentiated subjective responses observed between the two groups may be further interpreted in light of motor learning models applied to exercise training. According to a heuristic, participant-centered approach, motor experience is not limited to the mechanical execution of tasks but involves processes of exploration, adaptation, and autonomous construction of motor solutions. Within this framework, training can be viewed as an educational context in which individuals develop body awareness, self-regulation, and decision-making abilities [50].

From a practical perspective, these considerations highlight the importance of designing exercise programs that account not only for physiological responses but also for cognitive and perceptual processes underlying the exercise experience. In non-competitive and personal training settings, integrating training modalities that promote active engagement, task variability, and perceived challenge may enhance adherence, subjective well-being, and overall quality of the motor experience. Accordingly, a complementary use of HIIT and LISS within an individualized programming framework may represent an effective strategy to address both performance-related goals and psychological needs, improving the long-term effectiveness and sustainability of exercise interventions.

## 5. Conclusions

This pilot study indicates that both High-Intensity Interval Training (HIIT) and Low-Intensity Steady-State (LISS) exercise are effective strategies for improving body composition in physically active young adults, leading to significant reductions in fat mass, BMI, and body circumferences. Although no statistically significant between-group differences were observed for anthropometric variables, HIIT showed a more favorable tendency toward muscle mass preservation and greater perceived improvements in physical performance, whereas LISS was associated with greater improvements in self-reported psychological well-being.

Overall, the findings suggest that HIIT and LISS induce distinct but complementary adaptations. HIIT may be particularly suitable when training goals include time efficiency, performance enhancement, and maintenance of lean mass, while LISS represents a sustainable and psychologically rewarding option. Although the results cannot be generalized due to the pilot nature of the study, they provide useful indications for exercise programming and for the design of future research. Further studies involving larger samples, randomized allocation, and more precise assessments of body composition are warranted to confirm and expand upon the observed trends.

## Figures and Tables

**Table 1:** Parameters recorded for exercise intensity monitoring and protocol adherence.

Parameter	Unit	Collection Method	Frequency	Application
**Heart rate**	bpm	Polar H9 chest strap	Continuous	Intensity monitoring
% HRmax *	%	Individual calculation	Continuous	HIIT/LISS target zones
**Time in zone**	min	Polar Flow	Each session	Protocol adherence
**Estimated calories**	kcal	Polar algorithm	Continuous	Descriptive indicator

* HRmax: maximum heart rate.

**Table 2:** Example of a HIIT training session.

Phase	Exercise	Work Duration	Rest Duration	Intensity (%HRmax *)	Equipment	Notes
**WARM-UP**	Fast walking/stationary bike	10 min	—	50–60%	Treadmill/Bike	Activation
**CORE STABILITY AND CONTROL**		10 min				
**BLOCK 1**	Battle rope	30 s	30 s	80–95%	Battle rope	High intensity
**BLOCK 2**	Modified burpees	30 s	30 s	80–95%	—	Bodyweight
**BLOCK 3**	Kettlebell swing	30 s	30 s	80–95%	Kettlebell	8–16 kg
**BLOCK 4**	TRX Row	30 s	30 s	80–95%	TRX	Adjustable angle
**BLOCK 5**	Bike sprint	30 s	30 s	85–95%	Bike	Maximum intensity
**BLOCK 6**	Jump rope	30 s	30 s	85–95%	Rope	Cardiometabolic work
**RECOVERY BETWEEN ROUNDS**	—	60 s	—	60–70%	—	Total of 4 rounds
**COOL-DOWN**	Slow walking	10 min	—	50–60%	Treadmill	HR reduction

* HRmax: maximum heart rate.

**Table 3:** Example of a LISS training session.

Phase	Activity	Duration	Intensity (%HRmax)	Equipment	Notes
**Warm-up**	Light walking/elliptical	5 min	50–60%	Treadmill/Elliptical	Preparation
**Core stability and control**		10 min			
**Main phase**	Incline walking/Bike/Elliptical/Climber	40 min	60–70%	Treadmill, Bike, Elliptical, Climber	Steady intensity
**Cool-down**	Light walking	5 min	50–60%	Treadmill	Return to calm

HRmax: maximum heart rate.

**Table 4:** Training progression across the 12-week intervention.

Weeks	Frequency	Protocol	Objective	Notes
1–4	3 sessions per week	HIIT or LISS	Adaptation	Stabilization of intensity
5–8	3 sessions per week	HIIT or LISS	Consolidation	Slight progression
9–12	3 sessions per week	HIIT or LISS	Maximization of adaptations	Continuous HR monitoring

**Table 5:** Baseline characteristics of participants (T0) and between-group comparisons.

Variable	HIIT (T0)	LISS (T0)	Shapiro Wilk *p*	Test	*p* Value
Participants (n)	12	12			
Sex (M/F)	5/7	5/7			
Age (years)	30.42 ± 3.37	29.33 ± 2.56	0.171	Welch	0.393
Triceps skinfold (mm)	15.14 ± 6.50	14.47 ± 5.55	0.355	Welch	0.793
Subscapular skinfold (mm)	11.65 ± 4.16	13.72 ± 5.20	0.025	Mann–Whitney U	0.259
Biceps skinfold (mm)	5.98 ± 2.98	6.94 ± 3.60	0.017	Mann–Whitney U	0.521
Suprailiac skinfold (mm)	14.02 ± 5.53	15.57 ± 6.66	0.391	Welch	0.552
Supraspinale skinfold (mm)	11.35 ± 4.67	14.17 ± 7.00	0.074	Welch	0.275
Abdominal skinfold (mm)	19.11 ± 9.10	20.49 ± 6.58	0.152	Welch	0.679
Thigh skinfold (mm)	19.32 ± 6.02	19.32 ± 7.86	0.245	Welch	0.898
Calf skinfold (mm)	12.09 ± 4.98	13.03 ± 5.53	0.379	Welch	0.676
Arm circumference, relaxed (cm)	29.68 ± 2.82	30.77 ± 3.17	0.171	Welch	0.394
Arm circumference, flexed (cm)	30.53 ± 2.95	31.63 ± 3.56	0.248	Welch	0.428
Waist circumference (cm)	79.26 ± 12.68	79.99 ± 10.03	0.128	Welch	0.879
Hip circumference (cm)	97.98 ± 7.53	100.20 ± 6.21	0.283	Welch	0.446
Thigh circumference (cm)	49.92 ± 7.63	53.17 ± 2.73	<0.001	Mann–Whitney U	0.298
Calf circumference (cm)	36.42 ± 2.79	37.02 ± 2.97	0.127	Welch	0.618
Endomorphy	3.85 ± 1.27	4.25 ± 1.38	0.602	Welch	0.484
Mesomorphy	5.04 ± 1.17	6.76 ± 2.70	<0.001	Mann–Whitney U	0.977
Ectomorphy	1.66 ± 1.29	1.33 ± 0.90	0.084	Welch	0.487
BMI (kg/m^2^)	24.21 ± 3.69	25.48 ± 3.19	0.787	Welch	0.384

Values are mean ± SD unless otherwise stated. Between-group comparisons were performed using Welch’s *t*-test or Mann–Whitney U test as appropriate based on Shapiro–Wilk normality testing. *p*-values refer to HIIT vs. LISS at baseline.

**Table 6:** Descriptive changes in selected anthropometric variables across the intervention period (T0–T3).

Variable	HIIT T0	HIIT T1	HIIT T2	HIIT T3	LISS T0	LISS T1	LISS T2	LISS T3
**Body mass (kg)**	66.51	65.43	64.17	63.49	71.87	70.68	71.15	68.68
**BMI**	24.21	23.74	23.18	22.97	25.48	25.05	25.06	24.40
**Waist circumference (cm)**	79.26	77.58	76.14	75.44	79.99	78.63	78.89	76.78
**Hip circumference (cm)**	97.98	96.83	95.92	95.18	100.20	99.09	98.43	97.54
**Thigh circumference (cm)**	49.92	50.43	50.28	49.45	53.18	54.85	52.51	52.81
**Relaxed arm circumference (cm)**	29.68	29.43	29.28	29.19	30.77	30.61	30.63	29.93
**Endomorphy**	3.85	3.64	3.34	3.11	4.25	4.10	3.95	3.78
**Mesomorphy**	5.04	5.01	5.02	5.07	6.76	7.17	7.51	7.09
**Ectomorphy**	1.66	1.81	1.96	2.04	1.33	1.45	1.41	1.72

Values are mean values. T0: baseline; T1: 4 weeks; T2: 8 weeks; T3: 12 weeks; BMI: body mass index.

**Table 7:** Pre–post (T0–T3) changes in anthropometric variables in the HIIT group.

VARIABLE	MEAN T0	MEAN T3	DELTA	TEST	*p*-Value	SHAPIRO *p*
**BODY MASS**	66.51	63.49	−3.02	Paired *t*-test	0.0036	0.8667
**TRICEPS SKINFOLD**	15.14	10.88	−4.27	Wilcoxon test	0.0005	<0.001
**SUBSCAPULAR SKINFOLD**	11.65	9.79	−1.86	Paired *t*-test	0.0065	0.9486
**BICEPS SKINFOLD**	5.98	5.58	−0.40	Paired *t*-test	0.3276	0.2347
**ILIAC CREST SKINFOLD**	14.02	10.88	−3.14	Wilcoxon test	<0.001	0.0359
**SUPRASPINAL SKINFOLD**	11.35	9.33	−2.02	Paired *t*-test	0.0013	0.3881
**ABDOMINAL SKINFOLD**	19.11	14.75	−4.36	Paired *t*-test	0.0008	0.3171
**THIGH SKINFOLD**	19.32	15.96	−3.36	Paired *t*-test	0.0042	0.3404
**CALF SKINFOLD**	12.09	10.02	−2.07	Paired *t*-test	0.0147	0.6101
**RELAXED ARM CIRCUMFERENCE**	29.68	29.19	−0.48	Paired *t*-test	0.0943	0.5206
**FLEXED ARM CIRCUMFERENCE**	30.52	30.28	−0.24	Paired *t*-test	0.2577	0.9700
**WAIST GIRTH**	79.26	75.44	−3.82	Paired *t*-test	0.0003	0.7558
**HIP GIRTH**	97.98	95.18	−2.79	Paired *t*-test	0.0075	0.2761
**THIGH GIRTH**	49.92	49.45	−0.47	Wilcoxon test	0.1307	0.0026
**CALF GIRTH**	36.42	36.07	−0.35	Paired *t*-test	0.1076	0.0589
**FAT MASS [22]**	19.57	16.88	−2.69	Paired *t*-test	0.0004	0.9156
**MUSCLE MASS [23]**	25.67	26.14	+0.47	Paired *t*-test	0.0673	0.6265
**MUSCLE/BONE INDEX**	2.60	2.62	+0.02	Paired *t*-test	0.6181	0.0937
**ENDOMORPHY**	3.85	3.11	−0.75	Paired *t*-test	0.0002	0.5749
**MESOMORPHY**	5.04	5.07	+0.03	Paired *t*-test	0.4605	0.4786
**ECTOMORPHY**	1.66	2.04	+0.39	Paired *t*-test	0.0006	0.6708
**WAIST–HIP RATIO**	0.81	0.79	−0.01	Wilcoxon test	0.0106	0.0066
**CONICITY INDEX**	1.15	1.12	−0.03	Paired *t*-test	0.0003	0.1483
**BMI**	24.21	22.97	−1.24	Paired *t*-test	0.0004	0.4125

Values are means. Delta indicates mean change from T0 to T3. Normality was assessed using the Shapiro–Wilk test. Paired *t*-test or Wilcoxon signed-rank test was applied accordingly.

**Table 8:** Pre–post (T0–T3) changes in anthropometric variables in the LISS group.

VARIABLE	MEAN T0	MEAN T3	DELTA	TEST	*p*-Value	SHAPIRO *p*
**BODY MASS**	71.87	68.68	−3.19	Wilcoxon test	0.0034	0.0414
**TRICEPS SKINFOLD**	14.48	12.25	−2.22	Paired *t*-test	0.0027	0.3426
**SUBSCAPULAR SKINFOLD**	13.72	12.38	−1.35	Paired *t*-test	0.2143	0.1414
**BICEPS SKINFOLD**	6.94	5.88	−1.07	Wilcoxon test	0.0173	0.0038
**ILIAC CREST SKINFOLD**	15.57	13.29	−2.28	Paired *t*-test	0.0047	0.3680
**SUPRA-SPINAL SKINFOLD**	14.17	12.38	−1.79	Paired *t*-test	0.0140	0.7473
**ABDOMINAL SKINFOLD**	20.49	17.33	−3.16	Paired *t*-test	0.0001	0.9135
**THIGH SKINFOLD**	19.32	17.83	−1.48	Wilcoxon test	0.0655	0.0466
**CALF SKINFOLD**	13.02	12.25	−0.78	Wilcoxon test	0.1148	0.0041
**RELAXED ARM CIRCUMFERENCE**	30.77	29.93	−0.83	Paired *t*-test	0.0051	0.2369
**FLEXED ARM CIRCUMFERENCE**	31.63	30.83	−0.81	Paired *t*-test	0.0115	0.7509
**WAIST GIRTH**	79.99	76.78	−3.21	Wilcoxon test	0.0051	0.0067
**HIP GIRTH**	100.20	97.54	−2.66	Paired *t*-test	0.0287	0.9864
**THIGH GIRTH**	53.18	52.81	−0.37	Paired *t*-test	0.5550	0.2359
**CALF GIRTH**	37.02	36.92	−0.10	Paired *t*-test	0.7115	0.1191
**FAT MASS [22]**	21.30	19.61	−1.69	Wilcoxon test	0.0033	0.0160
**MUSCLE MASS [23]**	26.88	26.72	−0.16	Paired *t*-test	0.4799	0.7275
**MUSCLE/BONE INDEX**	2.65	2.76	+0.11	Wilcoxon test	0.8938	<0.001
**ENDOMORPHY**	4.25	3.78	−0.47	Paired *t*-test	0.0148	0.1086
**MESOMORPHY**	6.76	7.09	+0.33	Wilcoxon test	0.5337	<0.001
**ECTOMORPHY**	1.33	1.72	+0.39	Paired *t*-test	0.0169	0.0866
**WAIST–HIP RATIO**	0.80	0.82	+0.02	Wilcoxon test	0.1677	<0.001
**CONICITY INDEX**	1.12	1.18	+0.06	Wilcoxon test	0.1236	<0.001
**BMI**	25.48	24.40	−1.08	Paired *t*-test	0.0100	0.3345

Values are means. Delta indicates mean change from T0 to T3. Normality was assessed using the Shapiro–Wilk test. Paired *t*-test or Wilcoxon signed-rank test was applied accordingly.

**Table 9:** Between-group comparison of pre–post changes (Δ T3–T0) in anthropometric variables.

VARIABLE	Δ HIIT	Δ LISS	DIFFERENCE (HIIT–LISS)	TEST	*p*-Value	COHEN’S D
**BODY MASS**	−3.02	−3.19	0.18	Mann–Whitney U	0.84	0.06
**TRICEPS SKINFOLD**	−4.27	−2.23	−2.04	Mann–Whitney U	0.23	−0.55
**SUBSCAPULAR SKINFOLD**	−1.86	−1.35	−0.51	Welch *t*-test	0.65	−0.19
**BICEPS SKINFOLD**	−0.40	−1.07	0.67	Mann–Whitney U	0.46	0.49
**ILIAC CREST SKINFOLD**	−3.14	−2.28	−0.87	Mann–Whitney U	0.50	−0.33
**SUPRA-SPINAL SKINFOLD**	−1.31	−1.56	0.25	Mann–Whitney U	0.63	0.23
**ABDOMINAL SKINFOLD**	−3.67	−3.14	−0.53	Mann–Whitney U	0.71	−0.15
**THIGH SKINFOLD**	−2.61	−3.25	0.64	Welch *t*-test	0.48	0.31
**CALF SKINFOLD**	−2.56	−1.72	−0.84	Welch *t*-test	0.39	−0.36
**WAIST GIRTH**	−6.89	−5.72	−1.17	Mann–Whitney U	0.44	−0.29
**HIP GIRTH**	−4.02	−2.47	−1.54	Mann–Whitney U	0.32	−0.41
**THIGH GIRTH**	−0.94	−0.63	−0.31	Mann–Whitney U	0.68	−0.12
**CALF GIRTH**	−0.60	−0.45	−0.15	Mann–Whitney U	0.72	−0.10
**FAT MASS [22]**	−2.69	−1.69	−1.00	Mann–Whitney U	0.13	−0.58
**MUSCLE MASS [23]**	+0.47	−0.16	+0.63	Welch *t*-test	0.06	0.81
**ADIPO-MUSCULAR INDEX**	−0.05	+0.10	−0.15	Mann–Whitney U	0.28	−0.43
**MUSCLE/BONE INDEX**	+0.24	−0.07	+0.31	Welch *t*-test	0.15	0.52
**ENDOMORPHY**	−0.75	−0.47	−0.28	Welch *t*-test	0.21	−0.52
**MESOMORPHY**	+0.35	+0.29	+0.06	Mann–Whitney U	0.88	0.05
**ECTOMORPHY**	−0.28	−0.20	−0.08	Mann–Whitney U	0.75	−0.12
**WAIST–HIP RATIO**	−0.02	−0.01	−0.01	Mann–Whitney U	0.66	−0.18
**CONICITY INDEX**	−0.03	+0.06	−0.09	Mann–Whitney U	0.06	−0.47
**BMI**	−1.24	−1.09	−0.15	Welch *t*-test	0.72	−0.15

Values represent mean changes (Δ T3–T0). Between-group comparisons were performed using Mann–Whitney U test or Welch *t*-test, as appropriate. Cohen’s d represents effect size.

**Table 10:** Results of the mixed-design ANOVA (group × time) for selected anthropometric and somatotype variables.

VARIABLE	EFFECT	DF *	F	*p*	BRIEF INTERPRETATION
**BODY MASS**	Group	1, 9.24	0.53	0.484	No difference between groups
	Time	3, 20.93	9.51	<0.001	Significant decrease over time
	Group × Time	3, 52.58	0.31	0.819	No differential change between groups
**BMI**	Group	1, 8.66	0.30	0.597	No group differences
	Time	3, 20.97	11.15	<0.001	Significant reduction over time
	Group × Time	3, 52.02	0.48	0.695	Similar trajectories in both groups
**FAT MASS**	Group	1, 6.94	0.58	0.473	No group differences
	Time	3, 14.94	15.72	<0.001	Significant decrease over time
	Group × Time	3, 43.10	1.55	0.216	Comparable temporal change
**MUSCLE MASS**	Group	1, 9.96	0.21	0.661	No group differences
	Time	3, 16.92	0.28	0.840	No significant change
	Group × Time	3, 49.28	7.51	<0.001	Time-related change differs (HIIT preserves/increases muscle mass)
**ENDOMORPHY**	Group	1, 9.42	0.75	0.408	No difference between groups
	Time	3, 16.18	16.19	<0.001	Significant reduction in adiposity
	Group × Time	3, 51.64	1.61	0.197	Similar decrease in both groups
**MESOMORPHY**	Group	1, 9.97	1.37	0.270	No difference between groups
	Time	3, 22.12	0.30	0.828	No significant temporal change
	Group × Time	3, 52.72	1.34	0.272	No differential change
**ECTOMORPHY**	Group	1, 10.33	0.37	0.559	No difference between groups
	Time	3, 18.67	8.95	<0.001	Significant increase over time, reflecting greater linearity.
	Group × Time	3, 51.67	0.66	0.579	Similar temporal trend in both groups

* DF: degrees of freedom (Greenhouse–Geisser corrected where applicable). Significant *p*-values are reported for main effects of time and group × time interactions. Group: HIIT vs. LISS; Time: T0, T1, T2, T3.

**Table 11:** Between-group comparison of post-intervention subjective well-being and performance perceptions.

Variable (Item)	χ^2^	df	*p*	Cramer’s V	Interpretation
**D1—Psychological well-being (** **“** **I feel psychologically better compared to before the intervention.”)**	12.90	2	0.002	0.73	**Significant**; higher ratings in LISS
**D2—Motivation (“I experienced an increase in my motivation during the training program.”)**	1.67	3	0.644	0.15	Not significant
**D3—Physical well-being (“I perceive an improvement in my general physical well-being.”)**	4.39	2	0.111	0.30	Not significant
**D4—Functional capacity (“I feel more capable and functional in daily activities.”)**	3.95	2	0.139	0.29	Not significant
**D5—Overall performance (“I feel I have improved my overall physical performance.”)**	15.11	3	0.002	0.46	**Significant**; higher ratings in HIIT
**D6—Strength, speed, endurance (“I perceive myself as stronger, faster, or more enduring compared to before the intervention.”)**	20.67	3	<0.001	0.54	**Significant**; strong effect favoring HIIT

Between-group comparisons were performed using chi-square tests. Effect size was estimated using Cramér’s V. Higher values indicate stronger effects.

## Data Availability

The data presented in this study are available on request from the corresponding author. The data are not publicly available due to privacy and ethical restrictions.
